# Health-related quality of life and risk of colorectal cancer recurrence and All-cause death among advanced stages of colorectal cancer 1-year after diagnosis

**DOI:** 10.1186/1471-2407-14-337

**Published:** 2014-05-17

**Authors:** Carlos KH Wong, Wai-Lun Law, Yuk-Fai Wan, Jensen Tung-Chung Poon, Cindy Lo-Kuen Lam

**Affiliations:** 1 Department of Family Medicine and Primary Care, The University of Hong Kong, 3/F, Ap Lei Chau Clinic, 161 Ap Lei Chau Main Street, Ap Lei Chau, Hong Kong; 2 Department of Surgery, The University of Hong Kong, 2/F, Professorial Block, Queen Mary Hospital, 102 Pokfulam Road, Ap Lei Chau, Hong Kong

**Keywords:** Quality of life, Colorectal cancer, Prognosis, Survival, Recurrence, Competing risks

## Abstract

**Background:**

The study aimed to examine the association between health-related quality of life (HRQOL) assessed with overall survival (OS) and recurrence after diagnosis of colorectal cancer (CRC).

**Methods:**

Overall 160 patients with advanced stage CRC were recruited in an observational study and completed the generic and condition-specific HRQOL questionnaires at the colorectal specialist outpatient clinic in Hong Kong, between 10/2009 and 07/2010. Socio-demographic and clinical characteristics including duration since diagnosis, primary tumor location and treatment modality, were collected to serve as predictor variables in regression models. All-cause death or CRC recurrence was the event of interest. Association between HRQOL with OS was assessed using Cox regression. Association between HRQOL and CRC recurrence was further modeled by competing-risks regression adjusted for the competing-risks of death from any cause.

**Results:**

After a median follow-up of 23 months, there were 22 (16.1%) incidents of CRC recurrence and 15 (9.4%) deaths. Decreased physical functioning (hazard ratios, HR = 0.917, 95% CI:0.889-0.981) and general health of domains in SF-12 (HR = 0.846, 95% CI:0.746-0.958) or SF-6D scores (HR = 0.010, 95% CI:0.000-0.573) were associated with an increased risk of death, with adjustment of patients’ characteristics. Increased vitality (HR = 1.151, 95% CI:1.027-1.289) and mental health (HR = 1.128, 95% CI:1.005-1.265) were associated with an increased likelihood of death. In models adjusted for competing-risk of death, those with worse HRQOL was not associated with increased risk of CRC recurrence.

**Conclusions:**

Although self-reported HRQOL was not a significant prognostic factor for CRC recurrence, the HRQOL provided independent prognostic value about mortality in patients with advanced stage of CRC.

## Background

Colorectal cancer (CRC) is one of the most life-threatening cancers worldwide [1]. The estimated five-year relative survival rate of US patients with CRC was 69.5% for stage III and 11.3% for stage IV after adjustment for age [2]. The prognosis indicated by overall survival (OS) is primarily dependent on the stage of cancer. According to the American Joint Committee on Cancer (AJCC) classification system [3], the definition of advanced stage of cancer is when the carcinoma reaches any regional lymph nodes or has confirmed distant metastasis. In addition to clinical factors that influence the risk of mortality such as cancer stage [4,5], patients’ self-reported health-related quality of life (HRQOL) acts as the complementary biomedical indicator in clinical decision-making regarding the choice of therapeutic modalities due to the strong association between some aspects of HRQOL and OS [5-7].

Not only do the survivors of CRC patients at one year after diagnosis face the risk of mortality, but those with advanced stage of cancer also face the threat of cancer recurrence. However, whether the baseline HRQOL measure was associated with the risk of CRC recurrence remains uncertain and this measure was investigated in patients with breast cancer only [8,9]. To date, only one study investigated the associations between clinical characteristics and the risk of CRC recurrence after accounting for competing-risks of death from any cause [10], acknowledging that patients dying from any cause were no longer at risk of CRC recurrence other than censoring all-cause mortality in conventional approach. The aims of this study were to examine the association between HRQOL data and the risk of all-cause mortality using Cox regression approach, and the association between HRQOL and the risk of CRC recurrence using competing-risks regression approach in patients with advanced stage of CRC after controlling for the socio-demographic and clinical variables.

## Methods

### Subjects

This study was part of a sequence of studies using the health survey data to examine the HRQOL profile and health preference scores of Chinese patients with colorectal polyp/cancer [11-16]. Study data were retrieved from the 647 adult patients recruited at the colorectal specialist outpatient clinic of Queen Mary Hospital in Hong Kong between October 2009 and July 2010. Survival analysis was conducted to select the eligible patients who had advanced stages of colorectal cancer, stage III and IV as defined by AJCC cancer stage classification [3], at the initial diagnosis. Eligibility criteria of subjects were histologically confirmed by colorectal surgeons (WL Law and JTC Poon). Among 536 patients (response rate of 82.8%) completed the baseline survey, condition-specific Functional Assessment of Cancer Therapy-Colorectal (FACT-C) and generic SF-12 and SF-6D Health Survey were administered to 160 eligible patients (Stage III: 115, 71.9%; Stage IV: 45, 28.1%) by a trained research assistant after written consent was obtained. At baseline assessment, socio-demographic characteristics were obtained from the patients, whereas their clinical variables including duration since diagnosis of CRC, primary tumor location (Colon/Sigmoid vs Rectum), current CRC related treatment, cancer stage (Stage III vs Stage IV), presence of stoma (Permanent/Temporary vs Not even/Closure), and presence of prior CRC recurrence were retrieved from electronic medical record. Survival status was censored on the date of the last follow-up or on 14 August 2012.

Ethical approval was obtained from The University of Hong Kong/Hospital Authority Hong Kong West Cluster institutional review board (HKU/HA HKW IRB #UW 09–391), and this trial was registered with Hong Kong Clinical Trial Register (#HKCTR-973, Trial Registration Date: 6 Oct 2009) and Clinicaltrial.gov (#NCT02038283, Trial Registration Date: 28 August 2013).

### HRQOL measures

#### Functional assessment of cancer therapy-colorectal (FACT-C)

The FACT-C is a 34-item condition-specific HRQOL measure that covers a range of important aspects of quality of life in relation to patients with CRC. It assesses HRQOL across five subscales (physical well-being, PWB; social well-being, SWB; emotional well-being, EWB; functional well-being, FWB; and colorectal cancer subscale, CCS) each with five response options (“not at all”, “a little bit”, “somewhat”, “quite a bit”, and “very much”). The raw scores are computed to give standard scores in the possible range of 0–28 for PWB, EWB, FWB, and CCS subscales and 0–24 for SWB subscale. Higher subscale scores indicate better HRQOL. Previous studies have reported the psychometric properties regarding the validity and reliability of the instrument used in Chinese patients with colorectal neoplasms [15,17,18].

#### SF-12 health survey

The SF-12 is a widely used generic HRQOL measure assessing eight subscales (physical functioning, PF; role physical, RP; bodily pain, BP; general health, GH; vitality, VT; social functioning, SF; role emotional, RE; and mental health, MH). The theoretical range of the subscale and summary scores are from 0 to 100, with higher scores indicating better HRQOL. Psychometric properties of SF-12 have been examined in Chinese populations [19].

#### SF-6D health survey

The SF-6D is used as a generic preference-based measure assessing health across six dimensions (physical functioning, role limitations, social functioning, pain, mental health, and validity) with 3–5 response options. The theoretical range of SF-6D score is between 0.315 (worst possible health state) and 1 (perfect health) based on the Chinese Hong Kong scoring algorithm [20,21].

### Statistical analysis

Baseline socio-demographic and clinical characteristics of patients with different cancer stages (Stage III versus Stage IV) were compared using Chi-square test for categorical variables and Mann Whitney *U*-test for continuous variables. Mean scores and standard deviation of the scale in each instrument were calculated according to their official scoring algorithm. Since scoring algorithm of each instrument did not include guideline to deal with missing data, subjects with missing data in one of the instrument items were excluded in this study. Independent *t*-test was used to compare the differences in HRQOL scores between clinical characteristics. To retain the maximum information of the data, all HRQOL scores were analyzed with continuous nature of measurement rather than categorical variables [22].

Our event of interest was taken as 1) death from any cause or 2) CRC recurrence. Correspondingly, the primary objective of this study was to examine the associations between HRQOL, death and incidence of CRC recurrence accounting for the competing-risk of death from any cause. The survival duration was cumulated from the first month after completion of the survey. Each patient was observed from the date of recruitment to the study until the occurrence of CRC recurrence, death from any cause as the competing event, or date of last follow-up as censoring. Survival curves were estimated by Kaplan-Meier method and their differences between cancer stages were compared using the log-rank test. Univariate and multivariate Cox proportional hazards regression models were performed to estimate the associations between patients’ characteristics, represented by fixed-in-time variables, and HRQOL scores with the dependent variable of CRC recurrence or death from any cause. Three separate multivariate Cox models were fitted by assigning the independent variable of HRQOL as 1) FACT-C subscale scores, 2) the SF-12 domain scores, and 3) the SF-6D score. Hazard ratio (HR) and its 95% confidence intervals were reported for each variable in the regression models. Prior analysis alternatively modeled the effects of prognostic factors on all-cause mortality using competing-risks regression with censoring on recurrence [10]. However, the underlying assumption of random censoring was violated in such circumstance [10], and current analysis avoided the problem when the survived patients were censored at the time of loss to follow-up or data extraction date. Therefore, OS was defined as the time interval between the date when a patient was recruited to the study and the date of death from any cause/last follow-up.

Due to the fact that patients who died from any cause was impossible to have event of interest occurred, death from any cause acted as the competing event. To account for the competing-risks of death, competing-risks regression models based on Fine and Gray [23] were fitted to measure the association between patients’ characteristics and HRQOL on risk of CRC recurrence. This model further excluded 23 subjects who had a prior CRC recurrence before the baseline assessment. Sensitivity analysis was also conducted using conventional Cox regression analysis to examine the effect of patients’ characteristics and HRQOL on risk of CRC recurrence.

Predictive accuracy of Cox models was assessed and compared using Harrell’s discrimination *C*-index, ranging from zero to one. A value of 0.5 indicates no predictive discrimination, and values of 0 or 1.0 indicate perfect separation of subjects with different outcomes [24]. Goodness-of-fit for both Cox and competing-risk regression models were assessed using Akaike information criterion (AIC) and Bayesian information criterion (BIC). All regressions and other analyses were conducted using STATA version 12. In particular, competing-risks regression was estimated using STATA command stcrreg.

## Results

**Figure 1:**
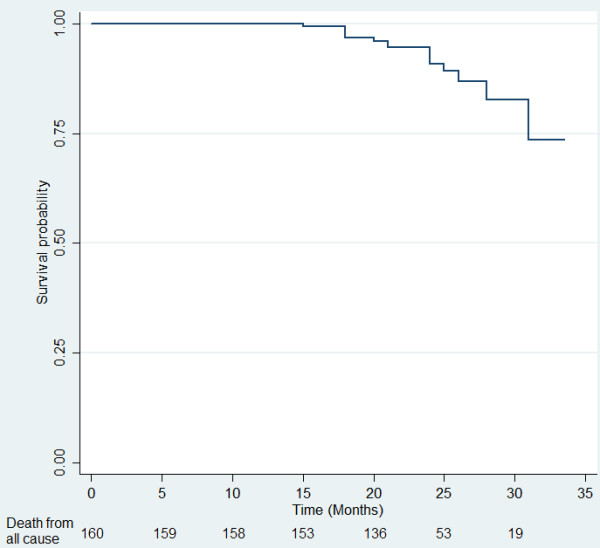
Overall Survival (months) of 160 colorectal cancer patients.

**Figure 2:**
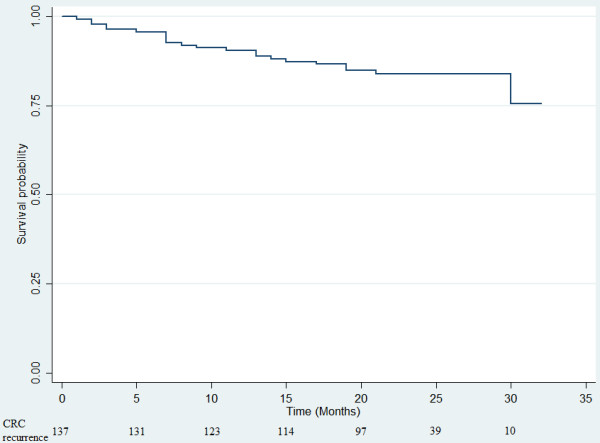
Colorectal cancer recurrence control (months) of 137 CRC patients without prior CRC recurrence.

At baseline, median age of patients was 62 years and 55.0% were male patients. The primary tumors were mostly (56.3%) located in colon. Among 160 patients with advanced CRC, there were 22 (16.1%) incidences of CRC recurrence and 15 (9.4%) deaths until the censored date in August 2012. Of those who died, there were 7 (50%) incidences of CRC recurrence between the baseline date and the death date. The majority (137, 85.6%) of patients with CRC did not have prior history of CRC recurrences whereas the remaining 23 patients did: 11 with liver metastasis, 2 with lung and liver metastasis, and 10 with other disease progressions. The survival distribution for the death from any cause and CRC recurrence are shown in Figures 1 and 2. Table 1 describes the baseline socio-demographic characteristics of patients with advanced CRC. Socio-demographic characteristics were compared between patients with different stages of CRC. Patients with stage IV CRC were more likely to be working-free or non-smokers than those with stage III CRC. Patients with stage IV CRC were more likely to be on CRC related treatment and recurrence than those with stage III CRC. Other socio-demographic and clinical characteristics were similar in patients with different cancer stages.

**Table 1:** Socio-demographic and clinical characteristics of patients with advanced stage of CRC

	**AJCC Stage classification**			
**Characteristics**	**Stage III (n = 115)**	**Stage IV (n = 45)**	**Total (n = 160)**	**P-value**
Age (Year,median, interquartile range)	62 (54–71)	62 (55–74)	62 (54–72)	0.301
Sex (%)				0.536
Male	56.5%	51.1%	55.0%	
Female	43.5%	48.9%	45.0%	
Education level (%)				0.939
No formal school	12.4%	11.1%	12.0%	
Primary	30.1%	26.7%	29.1%	
Secondary	43.4%	48.9%	44.9%	
Tertiary	14.2%	13.3%	13.9%	
Marital status (%)				0.942
Married	76.1%	75.6%	75.9%	
Not married	23.9%	24.4%	24.1%	
Currently working (%)				0.002^a^
Yes	29.2%	6.7%	22.8%	
No	70.8%	93.3%	77.2%	
Income (%)				0.075
≤HKD$20,000	78.4%	90.7%	81.8%	
>HKD$20,000	21.6%	9.3%	18.2%	
Smoking (%)				0.016^a^
Ever had	17.7%	35.6%	22.8%	
Never had	82.3%	64.4%	77.2%	
Drinking (%)				0.247
Ever had	26.3%	35.6%	28.9%	
Never had	73.7%	64.4%	71.1%	
Primary tumour site (%)				0.191
Colon	53.0%	64.4%	56.3%	
Rectum	47.0%	35.6%	43.8%	
Active CRC treatment (%)				<0.001^a^
No	80.7%	35.6%	67.9%	
Yes	19.3%	64.4%	32.1%	
Stoma (%)				0.112
Present	10.5%	20.0%	13.2%	
Absent	89.5%	80.0%	86.8%	
Time since diagnosis (month,median, interquartile range)	31 (13–59)	23 (11–52)	27 (12–58)	0.165
Vital status (%)				0.093
Alive	93.0%	84.4%	90.6%	
Death	7.0%	15.6%	9.4%	
Follow-up Month for Alive (median, interquartile range)	23 (21–25)	24 (23–26)	23 (22–25)	0.088
Follow-up Month for Death (median, interquartile range)	21 (18–24)	24 (18–26)	21 (18–25)	0.336
Follow-up Month for Total (median, interquartile range)	23 (21–25)	24 (22–26)	23 (21–25)	0.750
CRC recurrence^b^ (%)				<0.001^a^
Yes	10.2%	37.9%	16.1%	
No	89.8%	62.1%	83.9%	

Note: AJCC=American Joint Committee on Cancer.

^a^Significant differences (P<0.05) between groups by Mann-Whitney *U*-test or by Chi-square test, as appropriate.

^b^Excluded 23 subjects who had a prior CRC recurrence before the baseline assessment.

Table 2 presents the baseline HRQOL scores in patients with or without CRC recurrence, or who were alive or death till the end of study. Patients with lower role physical scores were more likely to have recurrent CRC (P = 0.006) whereas those with greater physical functioning, role physical or general health were more likely to stay alive (P = 0.004; P = 0.007; P = 0.011, respectively). There were no significant differences in all the five subscales of FACT-C instrument between vital status group and CRC recurrence group. The SF-6D score differed significantly in the alive and death patients (P = 0.019) but there was no significant difference between patients with or without CRC recurrence.

**Table 2:** Health-related Quality of Life in different levels of clinical characteristics

**Clinical characteristics**	**FACT-C**^**c**^					**SF-12**^**c**^								
	**PWB**	**SWB**	**EWB**	**FWB**	**CCS**	**PF**	**RP**	**BP**	**GH**	**VT**	**SF**	**RE**	**MH**	**SF-6D**^**c**^
Overall	25.2 ± 3.8	20.2 ± 4.2	21.0 ± 3.1	18.7 ± 4.0	21.6 ± 3.4	75.2 ± 29.9	72.5 ± 27.8	84.2 ± 25.2	52.2 ± 26.6	66.0 ± 17.9	77.2 ± 30.4	88.6 ± 19.9	80.1 ± 14.7	0.81 ± 0.13
AJCC Stage														
Stage III (n = 115)	25.5 ± 3.3	20.0 ± 4.4	21.1 ± 3.0	19.0 ± 4.1	21.3 ± 3.4	75.7 ± 30.5	74.2 ± 26.4	83.8 ± 25.9	52.4 ± 26.2	65.7 ± 17.8	78.8 ± 29.4	89.6 ± 18.3	80.1 ± 14.3	0.82 ± 0.13
Stage IV (n = 45)	24.5 ± 4.6	20.5 ± 3.8	20.9 ± 3.3	18.1 ± 3.9	22.3 ± 3.3	73.9 ± 28.7	68.6 ± 30.9	85.0 ± 23.5	51.8 ± 27.8	66.7 ± 18.5	73.3 ± 32.6	86.1 ± 23.3	80.0 ± 15.9	0.79 ± 0.14
P-value	0.142	0.536	0.720	0.238	0.123	0.733	0.264	0.792	0.899	0.767	0.313	0.321	0.964	0.225
Primary Tumour Site														
Colon (n = 90)	25.5 ± 3.4	20.5 ± 3.7	21.2 ± 3.0	18.6 ± 4.2	22.2 ± 2.9	77.6 ± 29.7	73.7 ± 26.6	84.6 ± 25.5	53.5 ± 28.4	67.2 ± 18.1	79.1 ± 29.4	87.8 ± 20.6	82.3 ± 13.2	0.81 ± 0.13
Rectum (n = 70)	24.9 ± 4.2	19.7 ± 4.8	20.7 ± 3.2	18.8 ± 3.8	20.9 ± 3.8	71.9 ± 30.0	70.9 ± 29.5	83.6 ± 24.9	50.5 ± 24.1	64.5 ± 17.7	74.6 ± 31.7	89.6 ± 19.1	77.1 ± 16.1	0.81 ± 0.13
P-value	0.346	0.214	0.296	0.809	0.023^a^	0.246	0.545	0.811	0.493	0.364	0.375	0.574	0.035^a^	0.873
Current Treatment														
Yes (n = 51)	25.9 ± 3.1	20.1 ± 4.0	21.0 ± 3.0	19.1 ± 3.9	21.8 ± 3.3	78.3 ± 30.5	76.4 ± 25.6	84.5 ± 24.0	55.6 ± 26.1	68.0 ± 16.7	82.0 ± 27.1	89.3 ± 18.0	80.0 ± 14.7	0.83 ± 0.12
No (n = 108)	23.9 ± 4.6	20.3 ± 4.7	21.0 ± 3.1	18.0 ± 4.3	21.2 ± 3.5	69.0 ± 27.9	64.8 ± 30.6	83.5 ± 27.5	45.4 ± 26.5	62.0 ± 19.7	67.5 ± 34.3	87.3 ± 23.3	80.3 ± 15.0	0.77 ± 0.14
P-value	0.003^a^	0.797	0.937	0.113	0.301	0.074	0.015^a^	0.819	0.026^a^	0.053	0.005^a^	0.564	0.922	0.010^a^
Stoma														
Yes (n = 21)	24.8 ± 3.1	20.2 ± 4.9	20.7 ± 3.8	17.2 ± 2.8	20.7 ± 4.4	71.3 ± 24.7	60.6 ± 27.3	75.0 ± 26.9	49.3 ± 24.3	61.3 ± 17.2	61.3 ± 31.9	89.4 ± 20.0	76.9 ± 19.6	0.77 ± 0.09
No (n = 138)	25.3 ± 3.9	20.1 ± 4.1	21.1 ± 3.0	18.9 ± 4.2	21.8 ± 3.2	75.8 ± 30.6	74.3 ± 27.5	85.6 ± 24.7	52.7 ± 27.0	66.7 ± 18.0	79.6 ± 29.5	88.5 ± 20.0	80.6 ± 13.8	0.82 ± 0.14
P-value	0.545	0.928	0.580	0.074	0.185	0.531	0.040^a^	0.080	0.596	0.204	0.011^a^	0.849	0.296	0.181
Vital Status														
Alive (n = 145)	25.3 ± 3.8	20.4 ± 3.9	21.0 ± 3.1	18.9 ± 4.1	21.6 ± 3.4	77.4 ± 29.0	74.4 ± 27.5	84.6 ± 24.5	54.0 ± 26.0	65.8 ± 17.4	78.3 ± 29.8	88.6 ± 20.4	79.7 ± 14.7	0.82 ± 0.13
Death (n = 15)	24.5 ± 3.6	18.1 ± 6.3	20.8 ± 2.9	17.2 ± 3.6	21.9 ± 2.9	53.6 ± 30.8	53.6 ± 24.2	80.4 ± 31.3	35.0 ± 27.0	67.9 ± 22.8	66.1 ± 34.8	88.4 ± 15.1	83.9 ± 15.1	0.73 ± 0.12
P-value	0.424	0.064	0.837	0.137	0.750	0.004^a^	0.007^a^	0.554	0.011^a^	0.685	0.152	0.970	0.306	0.019^a^
CRC Recurrence^b^														
Yes (n = 22)	24.3 ± 5.5	20.9 ± 5.1	20.2 ± 3.7	18.8 ± 3.1	22.0 ± 3.4	67.0 ± 27.2	57.4 ± 26.9	79.5 ± 29.5	51.8 ± 25.7	65.9 ± 23.8	68.2 ± 32.9	91.5 ± 19.0	83.5 ± 19.0	0.77 ± 0.13
No (n = 115)	25.6 ± 3.4	20.2 ± 4.0	21.2 ± 2.9	18.7 ± 4.4	21.4 ± 3.5	78.5 ± 29.2	75.0 ± 27.2	85.1 ± 24.6	53.4 ± 26.2	67.0 ± 16.7	80.2 ± 29.4	88.3 ± 20.0	80.0 ± 14.2	0.82 ± 0.13
P-value	0.151	0.501	0.149	0.925	0.449	0.091	0.006^a^	0.350	0.791	0.801	0.090	0.499	0.315	0.073

Note: AJCC = American Joint Committee on Cancer.

FACT-C subscales: PWB = physical well-being; SWB = social well-being; EWB = emotional well-being; FWB = functional well-being; CCS = colorectal cancer subscale;

SF-12 subscales: PF = physical functioning; RP = role physical; BP = bodily pain; GH = general health; VT = vitality; SF = social functioning; RE = role emotional; MH = mental health.

^a^Significant differences (P < 0.05) between groups by independent *t*-test.

^b^Excluded 23 subjects who had a prior CRC recurrence before the baseline assessment.

^c^Higher scores represent a higher level of functioning or a better HRQOL.

Table 3 demonstrates the results of univariate and multivariate Cox regression adjusted for socio-demographic and clinical characteristics. Univariate Cox regression indicated that a decrease in PF (HR = 0.975, 95% CI = 0.960-0.991, P = 0.003), RP (HR = 0.975, 95% CI = 0.956-0.994, P = 0.009), and GH (HR = 0.970, 95% CI = 0.948-0.992, P = 0.008) domains in SF-12 and SF-6D scores (HR = 0.007, 95% CI = 0.000-0.273, P = 0.008) was associated with poorer survival. All five FACT-C subscale scores and none of the socio-demographic and clinical characteristics at baseline were associated with OS. After adjustment for socio-demographic and clinical characteristics, the HR of five FACT-C subscale scores were also not significantly associated with OS with the C-statistic of 0.824 (95% CI = 0.681-0.968) but the statistical associations between HRQOL and all-cause mortality were significant in the SF-6D score (HR = 0.010, 95% CI = 0.000-0.573, P = 0.026) and some subscale scores of SF-12. The MH and VT subscales become significantly associated with OS after inclusion of HRQOL and other confounding variables, whereas the PF and GH were statistically significant in both univariate and multivariate analyses. The VT (HR = 1.151, 95% CI = 1.027-1.289, p = 0.015) and MH (HR = 1.128, 95% CI = 1.005-1.265, p = 0.040) were positively associated with the likelihood of death.

**Table 3:** Crude and adjusted hazard ratio (95% CI) of risk factors for death by univariate and multivariate cox regression analysis

	**Crude HR**^**a**^			**Adjusted HR (FACT-C domain)**^**a**^			**Adjusted HR (SF-12 domain)**^**a**^			**Adjusted HR (SF-6D)**^**a**^		
	**Estimate**	**95% CI**	**P-value**	**Estimate**	**95% CI**	**P-value**	**Estimate**	**95% CI**	**P-value**	**Estimate**	**95% CI**	**P-value**
Primary Tumour Site (Colon)												
Rectum	1.213	(0.438,3.361)	0.710	1.033	(0.233,4.589)	0.966	0.561	(0.054,5.855)	0.629	0.720	(0.212,2.454)	0.600
Active CRC Treatment (No)												
Yes	1.633	(0.569,4.682)	0.362	1.039	(0.195,5.529)	0.964	0.424	(0.052,3.432)	0.421	1.007	(0.239,4.243)	0.993
Stoma (Present)												
Absent	0.812	(0.181,3.644)	0.785	1.714	(0.212,13.840)	0.613	0.046	(0.000,4.874)	0.195	1.165	(0.189,7.168)	0.869
Time Since Diagnosis, months	0.993	(0.974,1.011)	0.427	0.998	(0.976,1.020)	0.839	1.015	(0.992,1.040)	0.199	1.003	(0.983,1.023)	0.801
AJCC (Stage III)												
Stage IV	1.898	(0.688,5.242)	0.216	1.928	(0.365,10.193)	0.440	2.791	(0.319,24.446)	0.354	1.659	(0.371,7.411)	0.508
FACT-C												
PWB	0.966	(0.862,1.083)	0.557	1.076	(0.857,1.352)	0.527						
SWB	0.908	(0.820,1.006)	0.066	1.011	(0.870,1.174)	0.889						
EWB	0.965	(0.825,1.129)	0.657	0.929	(0.683,1.264)	0.639						
FWB	0.905	(0.819,1.001)	0.053	0.902	(0.722,1.128)	0.365						
CCS	1.008	(0.862,1.179)	0.921	0.996	(0.796,1.247)	0.972						
SF-12												
PF	0.975	(0.960,0.991)	0.003^b^				0.917	(0.852,0.986)	0.019^b^			
RP	0.975	(0.956,0.994)	0.009^b^				0.996	(0.944,1.051)	0.882			
BP	0.994	(0.976,1.013)	0.552				1.055	(1.000,1.113)	0.052			
GH	0.970	(0.948,0.992)	0.008^b^				0.846	(0.746,0.958)	0.009^b^			
VT	1.005	(0.976,1.034)	0.754				1.151	(1.027,1.289)	0.015^b^			
SF	0.987	(0.972,1.003)	0.109				1.015	(0.970,1.063)	0.523			
RE	1.001	(0.975,1.027)	0.944				0.983	(0.919,1.051)	0.617			
MH	1.016	(0.975,1.058)	0.457				1.128	(1.005,1.265)	0.040^b^			
SF-6D	0.007	(0.000,0.273)	0.008^b^							0.010	(0.000,0.573)	0.026^b^
AIC				128.2			111.2			131.4		
BIC				187.3			179.8			179.1		
Harrell’s C-statistic				0.824 (0.681,0.968)			0.956 (0.909,1.002)			0.799 (0.678,0.921)		

Note: HR = Hazard ratio.

FACT-C subscales: PWB = physical well-being; SWB = social well-being; EWB = emotional well-being; FWB = functional well-being; CCS = colorectal cancer subscale;

SF-12 subscales: PF = physical functioning; RP = role physical; BP = bodily pain; GH = general health; VT = vitality; SF = social functioning; RE = role emotional; MH = mental health.

^a^HR > 1 indicates greater risk for death, with adjustment of socio-demographic characteristics.

^b^p-value < 0.05.

**Figure 3:**
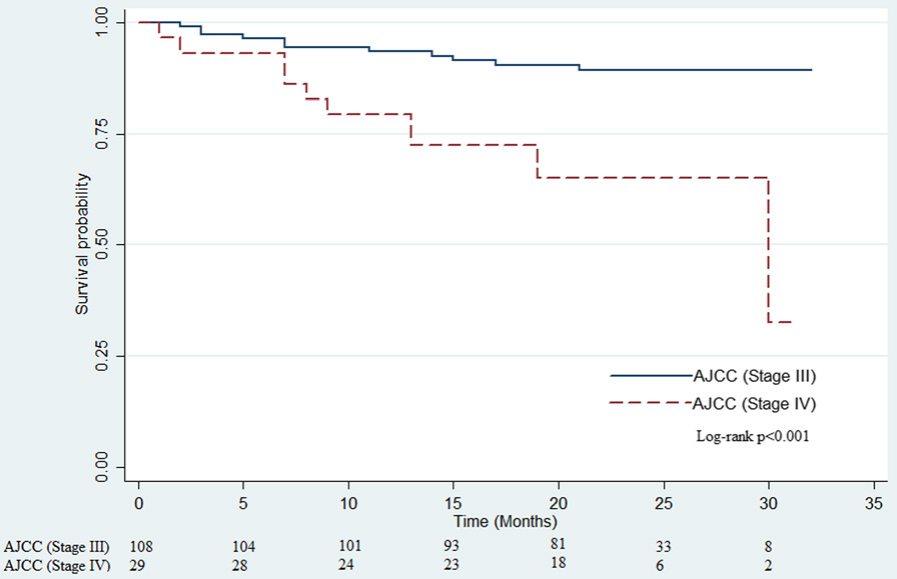
Colorectal cancer recurrence control (months) of 137 CRC Patients stratified by AJCC Stage (Stage III, n = 108; Stage IV, n = 29).

Table 4 shows the results of univariate and multivariate competing-risks regression adjusted for socio-demographic and clinical characteristics. Univariate analysis found that metastatic CRC, greater role functioning or SF-6D scores was associated with higher risk of CRC recurrence. After adjustment for socio-demographic and clinical characteristics, no measures of HRQOL were detected statistically significant. Figure 3 shows that patients with cancer stage III had significantly higher risk of CRC recurrence than those with cancer stage IV (log-rank test, p < 0.001). The sensitivity analysis found that the results of conventional Cox regression were similar to those of competing-risk regression (results not shown).

**Table 4:** Crude and adjusted hazard ratio (95% CI) of risk factors for CRC recurrence by univariate and multivariate competing-risks regression analysis

	**Crude SHR**^**a**^			**Adjusted SHR (FACT-C domain)**^**a**^			**Adjusted SHR (SF-12 domain)**^**a**^			**Adjusted SHR (SF-6D)**^**a**^		
	**Estimate**	**95% CI**	**P-value**	**Estimate**	**95% CI**	**P-value**	**Estimate**	**95% CI**	**P-value**	**Estimate**	**95% CI**	**P-value**
Primary Tumour Site (Colon)												
Rectum	1.173	(0.513,2.682)	0.705	1.084	(0.152,7.754)	0.936	1.419	(0.149,13.555)	0.761	1.121	(0.256,4.912)	0.880
Active CRC Treatment (No)												
Yes	3.891	(1.663,9.102)	0.002^b^	4.724	(0.812,27.477)	0.084	2.871	(0.824,10.006)	0.098	2.424	(0.771,7.617)	0.130
Stoma (Present)												
Absent	0.474	(0.179,1.254)	0.133	0.375	(0.078,1.814)	0.223	0.864	(0.145,5.159)	0.873	0.845	(0.205,3.484)	0.815
Time Since Diagnosis, months	0.984	(0.963,1.006)	0.152	0.998	(0.974,1.023)	0.892	1.002	(0.985,1.019)	0.817	0.992	(0.970,1.014)	0.481
AJCC (Stage III)												
Stage IV	4.002	(1.777,9.016)	<0.001^b^	2.469	(0.413,14.743)	0.322	6.225	(1.144,33.866)	0.034^b^	3.965	(1.222,12.862)	0.022^b^
FACT-C												
PWB	0.949	(0.890,1.011)	0.103	0.925	(0.765,1.119)	0.424						
SWB	1.042	(0.899,1.208)	0.583	1.067	(0.842,1.352)	0.593						
EWB	0.925	(0.831,1.029)	0.152	0.880	(0.702,1.102)	0.265						
FWB	1.001	(0.930,1.077)	0.988	1.022	(0.847,1.233)	0.821						
CCS	1.059	(0.921,1.217)	0.420	1.281	(0.980,1.674)	0.069						
SF-12												
PF	0.989	(0.978,1.000)	0.053				1.001	(0.970,1.033)	0.950			
RP	0.981	(0.968,0.994)	0.005^b^				0.972	(0.945,1.001)	0.058			
BP	0.993	(0.978,1.009)	0.398				0.992	(0.958,1.028)	0.663			
GH	0.998	(0.983,1.013)	0.770				1.002	(0.972,1.034)	0.877			
VT	0.998	(0.972,1.024)	0.852				1.009	(0.966,1.054)	0.690			
SF	0.989	(0.978,1.001)	0.070				1.008	(0.981,1.035)	0.569			
RE	1.008	(0.980,1.036)	0.579				1.036	(0.967,1.110)	0.319			
MH	1.015	(0.973,1.059)	0.478				1.028	(0.977,1.082)	0.286			
SF-6D	0.057	(0.004,0.888)	0.041^b^							0.503	(0.022,11.259)	0.665
AIC				187.4			202.5			199.4		
BIC				243.5			267.5			244.7		

Note: SHR = Sub-Hazard ratio.

FACT-C subscales: PWB = physical well-being; SWB = social well-being; EWB = emotional well-being; FWB = functional well-being; CCS = colorectal cancer subscale;

SF-12 subscales: PF = physical functioning; RP = role physical; BP = bodily pain; GH = general health; VT = vitality; SF = social functioning; RE = role emotional; MH = mental health.

^a^SHR > 1 indicates greater risk for CRC recurrence, with adjustment of socio-demographic characteristics.

^b^p-value < 0.05.

## Discussion

To the best of our knowledge, this was the first study to examine the association between HRQOL and the risk of mortality or CRC recurrence concurrently in patients with advanced stages of CRC. The prognostic value of HRQOL is considered important in predicting all-cause mortality. The Cox regression analysis found that SF-12 physical functioning, general health, vitality and mental health assessed at baseline were significantly associated with the risk of mortality. Fitting a survival model with clinical characteristics in conjunction with SF-12 data achieved better predictive accuracy and goodness-of-fit (C-statistic: 0.956; AIC: 111.2; BIC: 179.8) than models with FACT-C (C-statistic: 0.824; AIC: 128.2; BIC: 187.3) or SF-6D scores (C-statistic: 0.799; AIC: 131.4; BIC: 179.1). The use of competing-risks regression showed that no measures of HRQOL were associated with CRC recurrence, although univariate analyses detected statistically significant association in role functioning and SF-6D scores. The HRQOL data failed to improve the prediction of CRC recurrence. Despite insignificant associations between HRQOL and the risk of recurrence, incorporation of HRQOL assessments in routine clinical practice could provide important information about all-cause mortality in patients with advanced stage of CRC and have specialist outpatient consultation at one year after diagnosis.

Although the prediction of OS measured by FACT-C was not observed in this study, the majority of the existing literature reported the association between OS and HRQOL based on condition-specific measures developed by EORTC such as QLQ-C30 and QLQ-CR38. In previous studies of patients with any stages of CRC, strong evidence suggested that an increase in some functional scales measured by QLQ-C30 were associated with decreased risk of death in patients with CRC. Better social functioning score of QLQ-C30 was associated with improved OS among patients with metastatic colorectal cancer [6]. The prognostic value of social functioning was further confirmed by other validated samples from ten countries [25]. For metastatic patients treated with oxaliplatin-based first line chemotherapy, baseline physical functioning score was independently associated with the OS [26]. Baseline measurement of HRQOL including four functional scales (physical functioning, role functioning, social functioning and emotional functioning), global QOL and four symptom scales (nausea and vomiting, pain, dyspnoea and sleep disturbance) of QLQ-C30 had a significant association with the survival of patients with advanced CRC and at the commencement of receiving chemotherapy [5]. OS was predicted by the change in QLQ-C30 physical functioning, social functioning, appetite loss and global QOL scale scores from baseline to three months after treatment in the advanced stage of CRC [27]. For patients with rectal cancer, physical functioning, nausea and vomiting of QLQ-C30 and sexual enjoyment of QLQ-CR38 predicted the one-year survival after surgery [28]. In contrary to aforementioned studies, our findings found no association between the condition-specific measures and the risk of mortality or CRC recurrence.

Furthermore, the HRQOL assessed by non-EORTC instruments was found to be an independent predictor of survival. In a treatment clinical trial for patients with colorectal hepatic metastasis, longer OS was associated with higher physical scales of Rotterdam Symptom Checklist at baseline and lower deterioration in physical scales over time [29-31]. In an extension to all stages of colorectal cancer, health and physical subscales measured by the Quality of Life Index were prognostic measures of survival [32]. Meanwhile, pretreatment pain intensity scores measured by Brief Pain Inventory was a good prognostic factor of OS in patients with locally recurrent rectal cancer [7]. In line with previous studies using generic HRQOL measures, this study also found that greater physical functioning was associated with longer OS.

### Limitation

There were several limitations in this study. Firstly, the current study did not consider the regional lymph node ratio which was identified as one of the major prognostic factors in longitudinal studies [4,33-35]. Secondly, rates of all-cause mortality and recurrence reported in current study may be higher than those in other studies conducted in Chinese population [36,37], especially in studies of CRC patients undergoing curative open or laparoscopic resections. Patients in current study were recruited at specialist outpatient clinic where they were followed up at one year after diagnosis rather than a short period away from diagnosis or surgery. Thirdly, the total number of samples might be insufficient to check for internal validation by splitting baseline data into two or more portions. Apart from internal validation, future interesting work on an external validation utilizing an independent set of CRC data was warranted to strengthen the validity of model interpretation [22].

## Conclusion

To conclude, it was feasible to predict the OS through the assessment of generic HRQOL measure in the first year after diagnosis of advanced CRC. Although self-reported HRQOL was not a significant prognostic factor for CRC recurrence, the HRQOL measured by SF-12 instrument provided independent prognostic value and information about mortality in patients with advanced stage of CRC. This added-value of the HRQOL in conjunction with clinical characteristics may help in decision-making in clinical practice.

## Abbreviations

CRC: Colorectal cancer; HRQOL: Health-related quality of life; EORTC: European organization for research and treatment of cancer; QLQ-C30: Quality of life questionnaire core 30; FACT-C: Functional assessment of cancer therapy-colorectal; FACT-G: FACT-General; PWB: Physical well-being; SWB: Social well-being; EWB: Emotional well-being; FWB: Functional well-being; CCS: Colorectal cancer subscale; SF-12: Short Form-12; PF: Physical functioning; RP: Role physical; BP: Bodily pain; GH: General health; VT: Vitality; SF: Social functioning; RE: Role emotional; MH: Mental health; SF-6D: Short Form-6 dimensions; OS: Overall survival; HR: Hazard ratios

## Competing interests

The authors declare that they have no competing interests.

## Authors’ contributions

CW, CL initially conceived the study. All authors collectively designed and drafted the study protocol and sought funding and ethical approving. CW and YW led on statistical analyses. CW, WL, JP, CL contributed to recruitment and data collection. CW, YW, CL provided assistance with drafting and management protocol. CW and CL were the project coordinator, assisted with recruitment and coordinated the data collection. CW, YW, CL contributed to the drafting of the manuscript. CL is the PI of the funding application, coordinated the research network and research team. CW drafted the manuscript. All authors have read the draft critically and approved the final manuscript.

## Pre-publication history

The pre-publication history for this paper can be accessed here:

http://www.biomedcentral.com/1471-2407/14/337/prepub
